# Spinal Anesthesia and Minimal Invasive Laminotomy for Paddle Electrode Placement in Spinal Cord Stimulation: Technical Report and Clinical Results at Long-Term Followup

**DOI:** 10.1100/2012/201053

**Published:** 2012-04-01

**Authors:** S. Sarubbo, F. Latini, V. Tugnoli, R. Quatrale, E. Granieri, M. A. Cavallo

**Affiliations:** ^1^Division of Neurosurgery, Department of Neuroscience and Rehabilitation, University Hospital S. Anna, 203, C.so Giovecca, Ferrara, 44100 Ferrara, Italy; ^2^Section of Neurology, Department of Medical Surgical Sciences of Communication and Behaviour, University Hospital S. Anna, 203, C.so Giovecca, Ferrara, 44100 Ferrara, Italy; ^3^Division of Neurology, Department of Neuroscience and Rehabilitation, University Hospital S. Anna, 203, C.so Giovecca, Ferrara, 44100 Ferrara, Italy

## Abstract

*Object*. We arranged a mini-invasive surgical approach for implantation of paddle electrodes for SCS under spinal anesthesia obtaining the best paddle electrode placement and minimizing patients' discomfort. We describe our technique supported by neurophysiological intraoperative monitoring and clinical results. *Methods*. 16 patients, affected by neuropathic pain underwent the implantation of paddle electrodes for spinal cord stimulation in lateral decubitus under spinal anesthesia. The paddle was introduced after flavectomy and each patient confirmed the correct distribution of paresthesias induced by intraoperative test stimulation. VAS and patients' satisfaction rate were recorded during the followup and compared to preoperative values. *Results*. No patients reported discomfort during the procedure. In all cases, paresthesias coverage of the total painful region was achieved, allowing the best final electrode positioning. At the last followup (mean 36.7 months), 87.5% of the implanted patients had a good rate of satisfaction with a mean VAS score improvement of 70.5%. *Conclusions*. Spinal cord stimulation under spinal anesthesia allows an optimal positioning of the paddle electrodes without any discomfort for patients or neurosurgeons. The best intraoperative positioning allows a better postoperative control of pain, avoiding the risk of blind placements of the paddle or further surgery for their replacement.

## 1. Introduction

Spinal cord stimulation (SCS) is a widely established therapy for chronic neuropathic pain [1, 2]. This technique was proposed by Shealy [3] and is based on Melzack and Wall's gate-control theory [4]. According to this theory, the activation of large myelinated A-beta fibers inhibits the activity of small A-delta and C-fibers, carrying the pain sensation within the dorsal horns. Even if several mechanisms cooperate in pain relief provided by SCS, the activation of the A-beta fibers is considered to be the main mechanism [2, 5].

At the beginning, the stimulation was delivered using paddle electrodes inserted by means of a surgical operation, but in the early '70s percutaneous spinal catheter leads were developed. These two techniques are both still used, but with different indications. The percutaneous leads are less invasive and they are implantable under local anesthesia. The possibility to keep the patient awake enables the surgeon to verify the coverage of the painful areas “online”, asking directly the patient about the paresthesias distribution and thus achieving the optimal placement of the electrodes.

Anyway, paddle electrodes are, from several points of view, considered the best choice for patients who are responders to percutaneous SCS and for those who experimented catheter-leads displacement and/or misplacement. The shape of paddle electrodes offers a greater stability, decreasing the risk of migration [6, 7]. Moreover, the paddle electrodes cover a larger surface area and allow pain control with lower amplitude with respect to lead. It is attributable to their insulated backing, closer contact position to the dura, a greater cross-section of the electrodes, and more flexibility with programming. Finally, lower amplitudes also allow an effective battery energy saving with a crucial effect on health care costs. Paddle electrodes definitely provide better results of pain relief and, in these cases, more satisfaction for patients, with an improvement in their ability of everyday activities and a reduction of their analgesic drug intake [7].

The paddle electrode placement process is commonly performed under local anesthesia in order to obtain the best positioning. Unfortunately, this procedure often results very stressful and painful for patients [8]. Alternatively, some authors suggested performing this procedure under general anesthesia, predicting the resulting area of paresthesias using peripheral responses evoked by SCS. However, this technique has demonstrated to be unreliable [8, 9].

Other authors proposed spinal anesthesia to decrease the discomfort associated to the implantation under local anesthesia thus avoiding the risks of incorrect positioning of the paddle electrodes [8]. They obtained a complete motor block and anesthesia but not the block of all sensory transmission of the spinal dorsal column fibers. In this way, it was possible to identify the paresthesic area produced by SCS [8].

We adopted this technique, improving the minimal invasive spine approach. Clinical outcome over a long-term followup, surgical results, and details of our technique are here provided in order to suggest an original, reliable, and effective approach.

## 2. Patients and Methods

From January 2005 to February 2010, we selected 16 paddle electrode implantations under spinal anesthesia. All the patients have a history of neuropathic pain due to different causes, which irradiate to one or both inferior limbs, nonresponsive to the conventional therapies (14 failed back surgery syndrome; 1 multiple sclerosis; 1 luetic arachnoiditis) (Table 1) [7, 10]. All the patients, experimented the failure of conservative treatments: drug therapy (OTC Pain medication, NSAIDs, steroids, antiepileptic drugs, and opioids); injection therapy (local anesthetics and/or steroids); physical therapy. Finally, they resulted good responders to SCS with quadripolar or octopolar lead electrodes during the trial period, but after the permanent implantation they experienced multiple events of displacement or misplacement, with a nonoptimal distribution of paresthesias in the painful region. Considering the good response to SCS, we decided to propose to these patients the implantation of a plate electrodes system, in order to reduce the discomfort and the loss of effectiveness related to the periodic displacements. We implanted 8 pole paddle electrodes in 15 patients and a 16 pole paddle electrode in one patient affected by failed back surgery syndrome (FBSS).

For each patient the Visual Analogue Scale (VAS) score was recorded before surgery. Further assessment of VAS was obtained during the followup. Scores at the last followup were compared to preoperative results (mean followup 36,75 months; range 6–91). For each patient, the satisfaction rate was recorded during the follow-up period asking them to choose among little (0–50% pain relief), rather good (51–75%), good (76–90%), or total (91–100%). Finally, the rate of pain area coverage (total, subtotal, partial) was recorded during all the follow-up period to recognize possible displacement of the leads or malfunction of the implant.

All the patients subscribed a detailed informed consent, according to the procedure evaluated and accepted by the S. Anna University-Hospital Institutional Accreditation Committee and conformed to the standards set by the 1964 Declaration of Helsinki.

### 2.1. Technical Report

#### 2.1.1. Patient Preparation (See Video, Part I: Spinal Anesthesia, Lateral Position)

Intrathecal injection of hyperbaric Bupivacaine (15–20 mg) at the L2-L3 level was administrated for spinal anesthesia. The site of incision was deeply infiltrated with 10 cc of Mepivacaine (2% solution) in order to avoid postsurgical pain due to the scar tissue. The level of anesthetization of the region was careful assessed using traditional methods, such as pinch and pinprick.

#### 2.1.2. Positioning (See Video, Part I: Spinal Anesthesia, Lateral Position)

After spinal anesthesia administration, the patients were positioned on the operating table in lateral decubitus. The intervertebral space chosen for the electrode paddle insertion was evidenced under fluoroscopic guide (T8-T9 in 1 patient; T9-T10 in 2 patients; T10-T11 in 11 patients; T11-T12 in 2 patients) (Table 1).

#### 2.1.3. Surgical Technique (See Video, Part II: Flavectomy, Plate Electrode Introduction, Bone Anchoring)

We performed a median incision followed by bilateral skeletonization of the intervertebral space selected. After the resection of the supraspinous and interspinous ligaments, the upper spinous process was only partially resected. We did not provide any laminectomy, but we reached the epidural space by means of flavectomy. We introduced the paddle electrode into the epidural space and gently pushed it in a cephalad direction, by means of the guide.

#### 2.1.4. Stimulation Techniques (See Video, Part III: Neurophysiological Monitoring and Intraoperative Stimulation)

Once the paddle electrode was positioned into the epidural space, we connected the extremity of the external lead to the external test stimulator, by means of a screening cable. The test stimulation started and patients were asked to confirm the complete paresthesias coverage of the painful areas. The paddle position was eventually modified, according to the patients' sensations. Stimulation usually started with a gradual increasing of amplitude. We never needed amplitudes over 4 V to evoke paresthesias.

#### 2.1.5. Somatosensory and Motor Stimulation Techniques (See Video, Part III: Neurophysiological Monitoring and Intraoperative Stimulation)

In one case we also used intraoperative monitoring to evaluate the neurophysiological effects of spinal anesthesia and test stimulation.

Cortical-somatosensory-evoked potentials (SEPs) were obtained by stimulation of both tibial nerves of the ankle, alternately, via bipolar surface electrodes, and by bipolar stimulation from a spinal epidural grid (3 stimuli/sec, stimulus duration 0,2 ms, current intensity at motor threshold for peripheral and at sensory threshold for epidural stimulation). The cortical signals were continuously recorded by subcutaneous needle electrodes at the scalp in position Cz′ and at Fpz as reference (10–20 system). The potentials were filtered (bandpass 3 Hz–5 KHz), averaged (200 stimulus responses), and recorded for 100 msec after stimulus onset. Peripheral motor-evoked potentials (MEPs) and epidural-motor-evoked potentials (D-Wave) were obtained by magnetic motor cortex stimulation via eight coils positioned at the vertex of the scalp in the hot spot position and with adequate intensity for maximal responses of the tibial muscles. The epidural responses were collected by means of bipolar recordings via SCS grid. The peripheral signals were recorded by means of bipolar surface electrodes at the tibial muscle, bilaterally and continuously. The potentials were filtered (bandpass 3 Hz–5 KHz) and recorded for 50–100 msec after stimulus onset. All neurophysiologic data were performed with an electrodiagnostic system (Medtronic KeypPoint II, Minneapolis).

#### 2.1.6. Anchoring the Electrode Internally (See Video, Part II: Flavectomy, Plate Electrode Introduction, Bone Anchoring)

In our Institution, all the paddle electrodes were anchored perforating the spinous process of the inferior vertebra and securing the lead bodies to the spinous process, by means of 2-0 nonreabsorbable wires.

#### 2.1.7. Tunneling the Extensions

Further 5 cc of local anesthesia (Mepivacaine solution at 2%) was administrated along the tunneling route of the temporary extensions, approximately 3 cm below the surgical incision, also in this case to avoid postsurgical pain. The skin incision was then irrigated with Betadine-soaked gauzes and closed by layers, using absorbable sutures (Vicryl) for the subcutaneous tissues and nylon sutures for the skin.

#### 2.1.8. Implantation of IPG

The IPG position was chosen in agreement with the patients considering weight, fat distribution, and previous surgeries. However, we prefer to locate the pulse generator in an abdominal wound, especially in overweight patients.

A 5 cm skin incision is made and a subcutaneous pocket is created, above the muscle fascia. The IPG should not be deeper than 2 cm from the skin surface, especially if rechargeable IPG is used. In slim patients, a subfascial placement of the pulse generator may be desired for better cosmetic results. The leads were connected to the IPG, which was tested by external remote checking of the electrode impedances. The wound was then irrigated with Betadine-soaked gauzes and closed by layers using absorbable sutures for the subcutaneous tissue and nylon sutures for the skin. Appropriate dressings covered all the wounds.

## 3. Results

In all the procedures, analgesia was complete and effective, without interfering with the intraoperative monitoring of the paresthesia coverage.

As showed by neurophysiological monitoring (see Video, part 3), we observed the disappearance of short latency SEPs and cortical-evoked responses after tibial nerve stimulation, but we reported their preservation after intraoperative direct SCS somatosensory stimulation. This observation is also completed by the persistence of epidural D-waves after motor cortical stimulation and by the absence of peripheral motor responses after the same intraoperative motor cortex stimulation, as reported by other Authors, too [11].

The comparison between the preoperative and the last followup, Visual Analogue Scale (VAS) demonstrated a very good control of neuropathic pain by SCS in long-term periods. In our population, the mean VAS improvement was 70,5%, passing from a mean preoperative VAS value of 8,3 (range 7–10) to a mean postoperative value of 2,5 (range 1–7).

 Only two patients (no. 10, 11; Table 1) experienced scarce control of pain, respectively, with 22% and 50% of VAS improvement at the last followup.

8 patients (No. 1-2-3-7-9-13-14-15; Table 1) experimented a good rate of satisfaction in their pain relief (76–90%), 7 patients declared to be rather satisfied (51–75%), and only 1 patient (No. 10 Table 1) showed a scarce rate of pain relief (22%).

The pain area coverage had been demonstrated total for all the patients during intra-operative stimulation despite of variable distribution of neuropathic pain (5 patients with bilateral symmetric extremity pain, 3 patients with bilateral not symmetric extremity pain, 7 patients with a right or a left limb interested by pain, 1 patient experimented low back pain, and bilateral nonsymmetric extremity pain). During the follow-up period, patient No. 10 experimented a subtotal coverage of the pain area although radiographic imaging excluded displacement or breakage of the paddle electrode and checking impedances of circuit were also verified.

We had no complications in any but one patient, who developed a severe right leg paresis caused by an epidural hematoma that required an emergency intervention. After the hematoma evacuation, the patient recovered from all the symptoms and the paddle electrode was left in situ.

At the last followup no, patients needed new surgical procedures for miss or displacement of the electrode.

## 4. Discussion 

Chronic pain has an outstanding impact on a patient's quality of life. It interferes with his/her physical function and psychological well-being is often hindered. Moreover, chronic pain has been a well documented burden on our health care system, as well as on our social economy. Between 10 to 40% of patients who underwent lumbosacral spine surgery to alleviate pain, turned out to have persistent or recurrent chronic pain [12, 13], the so-called “failed back surgery syndrome” (FBSS). FBSS is the most common indication for SCS [10].

In selected patients with FBSS, treatment with SCS results in pain relief, sustained at a long-term followup and associated to patients' satisfaction and to important clinical improvements in their functional capacity and health-related quality of life [7, 14, 15]. Modern types of paddle electrodes enable steering of the paresthesias and provide a more stable lead position, resulting in a more consistent pain control.

The implantation of paddle electrodes requires a laminotomy and in many centers this procedure is performed under local anesthesia, which allows paresthesias coverage monitoring. This approach, however, is stressful, often painful and not well tolerated by some patients. Alternatively, the placement of such electrode systems can be performed under general anesthesia, definitely precluding the use of intraoperative test stimulation. The use of peripheral responses, evoked by SCS and interpreted as antidromic nerve activity, has been suggested to assess the optimal location of the electrode [16].

We experienced the use of spinal anesthesia for paddle electrode implantation with success, minimizing the discomfort for patients. The local anesthetic deposited into the subarachnoid space, in fact, produced complete motor block and anesthesia but did not seem to block all sensory transmission in the superficial layers of the spinal dorsal columns (as demonstrated by the integrity of the sensory cortical responses during intraoperative direct SCS somatosensory stimulation and by epidural D-wave persistence after cortical motor stimulation). Therefore, an intraoperative test of paresthesias distribution, guiding the lead positioning, is possible. Our data demonstrate that SCS can produce paresthesias for the intraoperative stimulation test even though spinal anesthesia coverage is completely effective in the control of pain. Our neurophysiological and clinical data seem to support a major involvement by anesthesia of the spinal roots and ganglia in respect to the coarse fibers of the dorsal columns [17]. Somatosensory-evoked responses, in fact, can be produced from segments below the anesthesia level, as also suggested by other authors [18–20].

The lateral position, turned out to be comfortable for awake patients and it does not increase the duration of surgery nor does it produce any kind of problems in gaining access to the spinal canal for surgeons. Furthermore, the minimal demolition we propose (strictly limited to flavectomy) allows the surgeon to avoid laminectomy, in order to minimize the risk of spinal instability also reducing the duration of surgery. This conservative surgical approach is reliable thanks to the flexibility of the plastic electrode. However, during flavectomy, much attention has to be paid to avoid the presence of bony splinters, so the paddle won't be damaged.

We are also used to anchor the lead bodies to the inferior spinous process in order to avoid the dislocation of the paddles. The effectiveness of this procedure is not largely approved but resulted effective to avoid displacements in our case series. In our study, the stimulation intensity required to evoke paresthesias during spinal anesthesia was only moderately higher than the amplitude needed in normal conditions. However, this difference from other studies on SCS under spinal anesthesia does not necessarily reflect a difference in the depth of analgesia, but may be the result of better electric conduction properties of the much larger and unidirectional stimulating poles of the paddle electrodes compared to catheter leads [8].

Our experience definitely suggests the safety and the effectiveness of the spinal anesthesia in providing the best positioning of electrodes for SCS. This kind of anesthesia provides a good analgesia without modifying the paresthesias perception, as showed in our case study. The opportunity to check the distribution of paresthesias in an intraoperative way allowed us the best positioning of the electrode; all our patients confirmed total pain area coverage during intraoperative stimulation. We have reached this goal, as confirmed by the good results in the control of pain carriers in our centre. The mean VAS improvement at long-term followup (36,7 months) remains remarkable (70,5%) with 87,5% of our population (14/16) characterized by a good rate of satisfaction.

Only two patients, in fact, did not reach a clinical success (defined as VAS improvement > 50%). One of them (No. 10, see Table 1) experimented a subtotal pain area coverage during the follow-up period even though radiological imaging excluded displacement of the paddle electrode. The other patient (No. 11, see Table 1), affected by multiple sclerosis, confirmed the total pain area coverage by paresthesias and he also refers to an important decrease of frequency of pain attacks, while the intensity of pain still remained high (VAS 5/10).

Nevertheless, the patient selection definitely remains the most important prognostic factor for good outcome, especially when considering the real etiology of neuropathic pain, the psychological status, and patients' compliance to this kind of treatment.

## 5. Conclusions

Our case series confirms the previous evidence about the safety and the effectiveness of spinal anesthesia for pain control, without creating any interference during intraoperative stimulation. The lateral position resulted comfortable for patients and feasible for surgeons. Flavectomy represents the most conservative surgical technique for the implantation of this kind of electrodes. Finally, we consider this procedure as the most effective in achieving a finer positioning of the paddle electrode, avoiding “blind placements”.

## Figures and Tables

**Table 1:** Summary of our case series. VAS (Visual Analogue Scale), FBSS (Failed Back Surgery Syndrome).

Patient No.	Gender	Age	Diagnosis	Symptoms	Level of implantation	Paddle electrodes	FollowUp Months	VAS Preopesation Postoperation	VAS % improvement	Rate of satisfaction	Adverse events
											
1	F	71	FBSS	Bilateral inferior limbs	T10-T11	8 poles	91	8-1	(87,5%)	Good	
2	F	64	FBSS	Bilateral inferior limbs	T11-T12	8 poles	76	9-2	(77%)	Good	
3	M	51	FBSS	Inferior left limb	T10-T11	8 poles	63	8-1	(87,5%)	Good	
4	M	39	FBSS	Bilateral inferior limbs	T9-T10	8 poles	61	7-3	(58%)	Rather good	
5	M	73	FBSS	Bilateral inferior limbs	T8-T9	8 poles	59	8-2	(75%)	Rather good	
6	F	70	FBSS	Bilateral inferior limbs (No symmetric)	T10-T11	8 poles	32	9-4	(56%)	Rather good	
7	F	37	FBSS	Bilateral inferior limbs	T10-T11	8 poles	29	8-1	(87,5%)	Good	Epidural hematoma
8	F	38	FBSS	Inferior left limb	T10-T11	16 poles	29	8-3	(62,5%)	Rather good	
9	F	76	FBSS	Bilateral inferior limbs (No symmetric)	T10-T11	8 poles	26	7-1	(85%)	Good	
10	M	40	FBSS	Inferior left limb	T9-T10	8 poles	25	9-7	(22%)	Little	
11	M	65	Multiple sclerosis	Low back pain + Bilateral inferior limbs (no symmetric)	T10-T11	8 poles	23	10-5	(50%)	Little	
12	F	56	FBSS	Inferior right limb	T10-T11	8 poles	20	8-3	(62,5%)	Rather good	
13	F	65	FBSS	Inferior left limb	T10-T11	8 poles	20	9-1	(88%)	Good	
14	F	58	Luetic arachnoiditis	Bilateral inferior limbs (no symmetric)	T10-T11	8 poles	16	9-1	(88%)	Good	
15	M	47	FBSS	Inferior right limb	T11-T12	8 poles	11	8-1	(87,5%)	Good	
16	M	67	FBSS	Inferior right limb	T10-T11	8 poles	6	9-4	(55%)	Rather good	
